# The serial mediation effect of perceived quality and customer satisfaction on the relationship between trust and repurchase intention: a research on private health insurance owners

**DOI:** 10.1186/s12913-025-12269-9

**Published:** 2025-02-15

**Authors:** İbrahim Gün, Selma Söyük

**Affiliations:** 1 Batman University, Batman, Türkiye; 2 Istanbul University-Cerrahpasa, Istanbul, Türkiye

**Keywords:** Trust, Perceived quality, Customer satisfaction, Repurchase intention, Private health insurance

## Abstract

**Background:**

This study examined the serial mediating roles of perceived quality and customer satisfaction in the relationship between trust and repurchase intentions among private health insurance owners.

**Methods:**

This cross-sectional study included 525 private health insurance owners. The data were collected between 15.12.2023 and 15.03.2024, and SPSS AMOS was used to analyze the direct and indirect estimates. The study utilized structural equation modeling (SEM) to examine the relationships among the constructs. The proposed model was tested using maximum likelihood estimation. Model fit indices and statistical significance levels were reported to ensure the robustness of the findings. Using an online survey, the participants completed self-reported measures of perceived quality, customer satisfaction, trust, and repurchase intention.

**Results:**

Trust significantly affected perceived quality, customer satisfaction and repurchase intention. Furthermore, perceived quality acted as a mediator in the relationship between trust and customer satisfaction. Additionally, customer satisfaction played a partial mediating role in the relationship between trust and repurchase intention. Both perceived quality and customer satisfaction play a serial mediating role in the relationship between trust and repurchase intention.

**Conclusions:**

This study highlighted the significance of perceived quality and customer satisfaction in the relationship between trust and repurchase intention. Private health insurance agencies both alleviate the burden on public health services and operate as profit-driven entities. Considering their indirect benefits to healthcare services, maintaining existing customer portfolios and acquiring new customers are important for both the health system and the profitability of insurance businesses.

**Supplementary Information:**

The online version contains supplementary material available at 10.1186/s12913-025-12269-9.

## Introduction

According to the data of the Insurance Association of Türkiye, the number of private health insurance policy owners was reported as 3,176,354 in the second quarter of 2023. This figure represents an increase of 291,068 policies compared with the same period in the previous year (2,885,286). In the first quarter of 2021, this number was 1,025,245 [1]. Considering official policy numbers, the private health insurance sector in Türkiye is growing rapidly. The key to success in the service sector lies in delivering high-quality services. Despite the widespread availability of numerous retail insurance options, customers may not be fully aware of the diverse range of choices [2]. Those who are aware of this situation also contemplate the intention to repurchase. Although the private health insurance sector in Türkiye demonstrates a growth trend, rising inflation and the increasing cost of living pose significant challenges for policyholders in renewing their insurance policies. According to the findings of a recent study conducted in Türkiye, a one-percentage-point increase in the inflation rate leads to a 0.72 percentage-point increase in real private health insurance premiums [3]. Consequently, policyholders aiming to renew their insurance policies may encounter difficulties in allocating the necessary financial resources amidst escalating living costs. However, trust in the insurance company, perceived quality, and customer satisfaction may positively influence policyholders’ willingness to renew their policies, enabling a more optimistic approach to resource allocation. In particular, companies in the growing private health insurance sector need to make greater efforts to retain their customers. Although the factors influencing customers’ repurchase intentions vary in the literature, customer satisfaction [4], trust in the insurance company [5] and perceived quality [6] are among the key factors that examined in this study.

Private health insurance plays a crucial role in alleviating the burden of healthcare expenses on the public sector, especially as approximately 80% of total healthcare expenditures in Türkiye are covered by the government. Approximately 47% of this expenditure is assumed by the Social Security Institution (SGK), whereas the remaining 32% is undertaken by the government [7]. The health insurance industry is broadly acknowledged as a key contributor to promoting public health and well-being [8]. Private health insurance is an elective form of insurance that offers coverage for individuals, facilitating the protection of their health and covering healthcare costs that may arise from encountered health risks [9]. Therefore, increasing the number of private health insurance policies plays a crucial role in reducing the burden on public healthcare services and decreasing public health expenditures.

Numerous studies have been conducted on the purchase behavior of healthcare services [10–13]. However, the number of studies in the field of private health insurance is quite limited. Specifically, there is a scarcity of research in the literature regarding the repurchase of private health insurance policies [2, 5, 14, 15]. We formulated the hypothesis that a serial mediating pathway exists, linking trust in the insurance company to repurchase intention through the mediating roles of customer satisfaction and perceived quality. To test this hypothesis, the present study employed a serial mediation model to investigate the relationship between trust and repurchase intention within the private health insurance sector. This approach provides a novel framework for analyzing the interplay of multiple variables in the relationship. Unlike parallel mediation models, which assume that mediators do not exert causal effects on one another, serial mediation accommodates the possibility of a causal sequence among mediators. This methodological advantage enables the exploration of specific theoretical pathways and their relative significance within a cohesive model. To the best of our knowledge, private health insurance in Türkiye is being analyzed for the first time using multiple variables, considering both direct and indirect effects, and it makes significant contributions to the literature. Establishing a strong presence in the growing sector and retaining existing customers is crucial. This study is the first in Türkiye to address trust, perceived service quality, and customer satisfaction, which are vital for ensuring customer loyalty. The number of publications on private health insurance in Türkiye is very limited, and no studies have been found that investigate repurchase intention in private health insurance companies. In the international literature, these concepts have also been underexplored within the private health insurance sector. Therefore, we believe that this study will play a pioneering role in filling a gap in the existing literature.

## Literature review and hypotheses development

The repurchase intention is associated with the theory of status quo bias theory and the disconfirmation theory. According to the theory of status quo bias, consumers tend to avoid the risk of changing or switching service providers. Therefore, consumers with a status quo bias exhibit positive value, information, and emotional attachment despite lucrative offers from competitors or pressures for change. This significantly influences the intention to repurchase [2]. On the other hand, disconfirmation theory seeks to understand how the disparity between a consumer’s expectations and experiences influences consumer satisfaction and perceptions of a product or service [16]. Furthermore, in many countries, individuals can often purchase supplementary insurance that covers illnesses with high costs and low probabilities. The evidence presented by the authors indicates that in these situations, individuals assign different weights to probabilities [17]. This research has contributed to the theories and tested their validity within the context of private health insurance.

### Repurchase intention

The consumers’ intent to repurchase a product or service depends simply on their past purchase experiences. Consumers can possess positive and negative repurchase intentions on the basis of their attitude toward service performance [10]. Repurchase intention has been described as “Customers’ anticipation of purchasing again, their commitment to retain the relationship or intent to continue a relationship with a provider for the foreseeable future” [18].

When factors influencing repurchase intentions are examined, the most studied variables are as follows: service convenience [10], satisfaction [19], perceived quality [20], perceived value [21], trust [22], and word of mouth [23]. Furthermore, according to Saraswati and Rahyuda [24], perceived ease of use, usefulness, and trust significantly influence repurchase intention.

### Customer satisfaction

Compared with other sectors, such as business, education, and tourism, the healthcare insurance industry's customer satisfaction is relatively understudied, despite the substantial size and volume of operations in this specific industry [25]. Satisfaction with an object, in this case health insurance, is influenced by the level of interaction between users/service seekers and service providers [26]. In this context, customer satisfaction can be defined as the perception of a satisfactory level of fulfillment derived from the experience with healthcare insurance [25]. In the literature, customer satisfaction is defined as the result of assessing the dissonance between customers’ expectations and the real experiences derived from the use of goods and services [27].

Satisfaction with a product or service is of critical importance in determining the intention to repurchase. Consumer satisfaction is also a significant component. Satisfaction is the feeling of happiness or disappointment that arises after perceptions or expectations are compared with the performance (results) of a product. Satisfaction is a customer's response to a discrepancy between the previous level and the actual performance felt after usage [28]. Customer satisfaction is the most important factor influencing customer loyalty in supplemental health insurance [29]. Nevertheless, client satisfaction is necessary but not sufficient to influence repurchase intention [19]. On the basis of the evidence in the literature, we examine the effects of other factors on repurchase intentions in the present study.

### Trust

In a healthcare system with a competitive environment, citizens are expected to be critical consumers when choosing a health insurance policy, and the choice of a health insurance policy may be related to trust in the health insurer [15]. Trust is a psychological construct that involves the judgments of individuals or groups regarding the belief that systems or individuals within a system will be effective and results oriented toward their needs [26].

Trust is very important in health care, not only between patients and physicians but also between patients and medical institutions such as hospitals and health insurers [30]. Trust is a relational notion: it generally lies between people, people and organizations [31]. Trust was defined by FJ van der Hulst et al. [15] as “the optimistic acceptance of a vulnerable situation in which the truster believes the trustee will care for the truster’s interests.” Furthermore, RE Bes et al. [30] defined trust as “the optimistic acceptance of a vulnerable situation where the trustor believes that the trustee has his best interests at heart.”

Trust has previously been investigated in the context of health insurers. In this context, the results indicate, for example, that trust in the health insurer is positively correlated with a reduced inclination to switch insurers and a lower incidence of disputes with the insurer [30]. Customers show a status quo bias. Trust, positive communication, satisfaction and perceived quality help customers repurchase insurance policy [32].

Previous studies have shown that trust significantly affects customer satisfaction [33] and repurchase intentions [34, 35] and is positively associated with perceived quality [28]. Therefore, one of the aims of the present study was to investigate the effect of trust on repurchase intention through customer satisfaction and perceived quality.

### Perceived quality

Hellier et al. [36] defined perceived quality as “the customer’s overall assessment of the standard of the service delivery process.” Based on previous studies, perceived quality is a determinant of customer satisfaction [22, 37], perceived value and equity [36], behavioral intention [38], and repurchase intention [35, 39].

The available evidence regarding the correlation between enrollment in health insurance and the perceived quality of healthcare is quite limited [40]. Similarly, the impact of trust on repurchase intentions in the insurance sector is also limited.

Customers possessing information about prices may exhibit varying degrees of price sensitivity. A reduced sensitivity to price may be linked to elevated perceptions of higher quality, where customers anticipating superior quality tend to demonstrate lower price sensitivity. Moreover, an actual experience of higher quality can result in a diminished sensitivity to price [28]. Therefore, insurance companies face pricing challenges. PV Rosenau and CJ Lako [41] stated that the Dutch health insurance model may not control costs, public satisfaction is low, and perceived quality is low and may not be as effective as expected in the United States. In Türkiye, the number of studies conducted in the health insurance industry is quite limited, and to the best of our knowledge, no study has investigated the effect of perceived quality on repurchase intentions.

## Interrelationships among trust, perceived quality, customer satisfaction, and repurchase intention

Perceived quality and trust are significantly related variables. Trust in an insurance company plays a crucial role in influencing how customers perceive the quality of the services offered [37]. A previous study indicated that the perceived quality of an online store’s website is directly related to users’ trust [42]. On the basis of the literature, the following hypothesis was generated:

- H_1_: Trust is significantly related to perceived quality.

Trust is a determinant of customer satisfaction [33]. According to previous studies in the literature, perceived service quality significantly impacts customer satisfaction [43, 44]. Trust seems to be the most influential dimension for consumer satisfaction in the proposed model by authors [45]. Moreover, trust and customer satisfaction are significantly and positively related to customer loyalty, which in turn results in repurchase intention [46]. On the basis of the literature review, the following hypothesis was formulated:

- H_2_: Trust is significantly related to customer satisfaction.

The impact of trust on repurchase intentions has been investigated in various fields, and the literature indicates a positive and significant effect [34]. A study conducted by L Xiao et al. [35] reported that consumer repurchase intentions are influenced by trust. In the e-commerce sector, trust has a significant and positive effect on repurchase intentions, similar to the impact of perceived value [39]. A recent study conducted in China indicated that, trust positively affect users’ repurchase intention [47]. In another fresh study has demonstrated the significant effects of brand trust on repurchase intention [48]. Additionally, on the basis of disconfirmation theory, if there is a discrepancy between a customer’s expectations and the outcomes they experience, feelings of dissatisfaction may arise. In such cases, the customer may develop a negative sentiment towards the service provider, which can subsequently affect their repurchase intention in a negative way [16]. On the basis of studies in the literature, the following hypothesis has been developed:

- H_3_: Trust is significantly related to repurchase intentions.

The effect of perceived quality on customer satisfaction has been investigated in many studies. A summary of the literature indicates that perceived service quality positively affects customer satisfaction [37]. Many studies have shown that perceived service quality has a significant effect on customer satisfaction [5, 20, 22]. A novel study stated that, service quality significantly influences customer satisfaction with health insurance in Saudi Arabia [49]. On the basis of studies in the literature, the following hypothesis has been developed:

- H_4_: Perceived quality is significantly related to customer satisfaction.

The impact of customer satisfaction on repurchase intention has been investigated in various fields, and the literature indicates a positive and significant effect [5, 50, 51]. Studies have shown that satisfied customers tend to repurchase more [52]. Furthermore, satisfied consumers are more likely to continue their relationship with a firm than dissatisfied consumers are [53]. The customer satisfaction and loyalty theory propose that the perceived quality of service by users influences their attitudes and behaviors over the long term [54]. Moreover, customer satisfaction customer satisfaction had a significant positive effect on patronage [5] However, it should be noted that, in addition to customer satisfaction, other factors influence repurchase intention, as stated above. Owing to the limited number of studies in the field of private health insurance, this study aims to examine the effect of customer satisfaction on the repurchase intention of private health insurance policies. On the basis of these explanations, we proposed following hypothesis:

- H_5_: Customer satisfaction is significantly related to repurchase intentions.

The customer satisfaction and loyalty theory suggest perceived quality is related to customer attitudes and behaviors [54]. A recent study’s findings stated that the perceived quality affects the perceived value as well as customer satisfaction and repurchase intention [55]. In this regard we not only investigated direct relationship between customer satisfaction and repurchase intention, but also mediating role of customer satisfaction in the relationship perceived quality and repurchase intention. On the basis of this aim, we proposed following hypothesis and hypothesis 8:

- H_6_: Perceived quality is significantly related to repurchase intentions.

## Mediating effect of perceived quality and customer satisfaction

Customer repurchase intention is influenced by various factors, including service quality, perceived value, and overall satisfaction, with customer satisfaction playing a critical mediating role in this process [51, 56]. The relationship between service quality and repurchase intention is shaped through perceived quality, which positively impacts satisfaction and, in turn, drives repurchase intention [57, 58]. Satisfaction is a key factor in fostering service loyalty, as it mediates the connection between perceived service quality and both repurchase intention and customer loyalty [59, 60].

Perceived quality and customer satisfaction are both essential elements that influence repurchase intention and customer loyalty. Satisfaction often mediates the effect of service quality on customer behaviors such as repurchase, which has been supported by a range of studies [46, 59, 60] For instance, in the tourism sector, satisfaction fully mediated the relationship between perceived service quality and repurchase intention [61], and similarly, in other sectors, satisfaction mediated the effects of perceived service quality and brand image on repurchase intention [62]. In the health insurance industry, customer satisfaction plays a mediating role between perceived value and repurchase intention, while trust in the service provider moderates this relationship [5]. Trust further enhances the perception of service quality, boosting customer satisfaction and loyalty, which ultimately leads to increased repurchase intention [37]. This suggests that trust indirectly influences repurchase intention by first improving both perceived quality and satisfaction. Based on these findings, we propose the following hypotheses:

- H_7_: Perceived quality has a mediating role in the relationship between trust and satisfaction.
- H_8_: Customer satisfaction has a mediating role in the relationship between perceived quality and repurchase intention.
- H_9_: Perceived quality and customer satisfaction play serial mediating roles in the relationship between trust and repurchase intention.

**Fig. 1:**
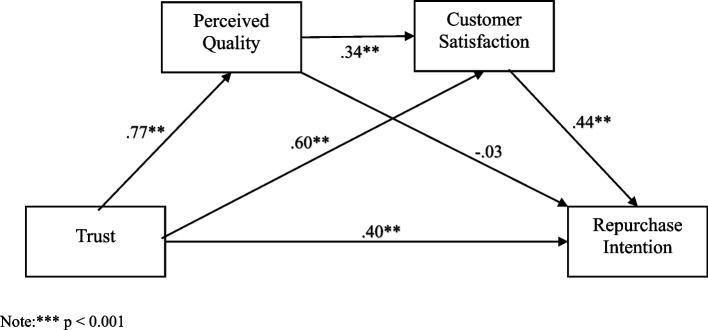
The proposed structural model. Note:*** *p* < 0.001

**Fig. 2:**
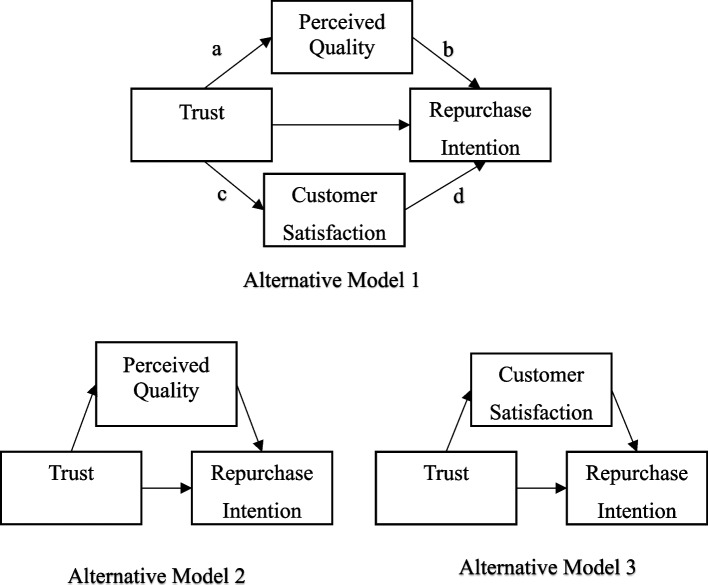
Alternative models

This study employs a serial mediation model to explore the interplay between trust, perceived quality, satisfaction, and repurchase intentions. This innovative method offers a new perspective for examining the interplay of multiple variables within this relationship. While parallel mediation models assume that no mediator exerts a causal influence on another, such an assumption does not hold in the case of serial mediation. This unique feature enables the analysis of a particular theoretical sequence among the variables. As a result, this statistical method provides the benefit of evaluating multiple pathways between the variables and determining their significance within an integrated model. Additionally, it allows for the examination of the role of the variable sequence in a manner consistent with the theoretical framework underpinning the study. To the best of our knowledge, few studies have explored factors influencing repurchase intentions in private health insurance, and none have examined the combined mediating effects of perceived quality and customer satisfaction in the trust-repurchase intention relationship. The proposed structural model is depicted in Fig. 1, while alternative models are shown in Fig. 2 for comparison.

## Methods

### Participants

The participants of the present study consisted of 525 individuals who voluntarily purchased private health insurance in İstanbul. When the ages of the participants were examined, those aged 35 years and above accounted for approximately 60% of the sample. Furthermore, 62.3% of the participants were women. In terms of education level, the largest group was composed of graduates, accounting for 61.1% of the sample. Approximately 79% of those supporting the research reported having no chronic illnesses. Among the policyholders, 29.1% indicated that they had never used their policy in the past year, whereas approximately 35% reported using it 1–3 times per year. A little over half of the policyholders mentioned that they had owned private health insurance policies for 1–7 years, and approximately 39% had supplementary types of policies. A full description of the participants is presented in Table 1.

**Table 1:** Full description of demographic variables

Variable	Level	N	%
Age	19–24	84	16.0
	25–34	120	22.9
	35–44	108	20.6
	45–54	111	21.1
	55 and more	102	19.4
Gender	Female	327	62.3
	Male	198	37.7
Education	High School	45	8.6
	Undergraduate	321	61.1
	Graduate Degree	159	30.3
Chronic disease	Yes	111	21.1
	No	414	78.9
Time of use private health insurance in a year	Never	153	29.1
	1–3 times	183	34.9
	4–6 times	129	24.6
	7–10 times	45	8.6
	11 and more	15	2.9
Length of time owing private health insurance	Under 1 year	93	17.7
	1–3 years	165	31.4
	4–7 years	126	24.0
	8–15 years	84	16.0
	16 years and more	57	10.9
Policy type	Outpatient	135	25.7
	Inpatient	180	34.3
	Supplementary	204	38.9
	Emergency care	6	1.1
	Total	525	100.0

## Measures

### Trust scale

Trust to the Insurance Company scale was developed by CM Chiu et al. [63] and was adopted for the insurance market by the authors. The scale has a single structure that is rated on a 5-point Likert-type scale (1 = strongly disagree; 5 = strongly agree). It includes 5 items, and a sample item is “On the basis of my past experience, I know that the company I purchased the policy from is honest.” The composite reliability of the scale was 0.96. The factor loads of the present study ranged from 0.69 to 0.88, and the Cronbach’s alpha was 0.90. A high score obtained from the scale indicates a high level of trust in the insurance company. English version of the scale presented in Appendix A.

### Perceived quality scale

The perceived quality scale measures a customer’s overall assessment of the standard of the service delivery process. In this context, the scale aimed to examine the quality of the services offered by the insurance company. The scale was developed by Hellier et al. [36] and validated by authors in the Turkish language. The scale has a single structure that is rated on a 5-point Likert scale (1 = Strongly disagree; 5 = Strongly agree). Perceived quality was measured with 5 items, and a sample item is “The insurance company employees (agent) tell me exactly when the health services will be performed.” In the present study, factor loads ranged from 0.73–0.85, and the Cronbach’s alpha value was 0.97. English version of the scale presented in Appendix A.

### Customer satisfaction scale

The customer satisfaction scale was adopted for the insurance market by Hellier et al. [36] to measure satisfaction with insurance policy. The scale has a single structure, and 4 items are rated on a 5-point Likert scale (1 = Strongly disagree; 5 = Strongly agree). A sample item is “My decision to purchase health insurance from the company was a wise one.” In the present study, factor loads ranged from 0.77 to 0.94, and Cronbach’s alpha was 0.93. A high score indicates high satisfaction with private health insurance. English version of the scale presented in Appendix A.

### Repurchase intention scale

The scale was developed by JI Shin et al. [64] and adopted into Turkish validation conducted by Gün [65]. The scale has a single structure that includes 3 self-reported items that are evaluated using a 5-point Likert-type scale (1 = strongly disagree; 5 = strongly agree). The factor load ranged from 0.84 to 0.94. A sample item is “I will continuously obtain my health insurance from the same insurance company.” In this study, the Cronbach’s alpha was 0.92. A high score obtained from the scale indicates a high level of repurchase intention related to the insurance policy.

### Procedure

A pilot study was conducted with 50 private health insurance policy holders. The language validity of the scales was assessed, and the scales were prepared for use through factor analyses. The research population comprised individuals who hold private health insurance policies in İstanbul, Türkiye. In the present study convenience sampling method was used. Due to the restrictions imposed by data protection laws, insurance companies offering private health policies are prohibited from sharing their customers’ information with third parties. This limitation makes accessing policyholders challenging, resulting in significant time and cost inefficiencies. Consequently, the convenience sampling method was employed as it provides a practical and cost-effective approach under these constraints. Ethical considerations, such as the protection of personal data, further restrict the applicability of probability-based sampling methods like random or stratified sampling. While the limitations of convenience sampling, such as reduced generalizability, are acknowledged, it was deemed the most feasible option to ensure the study’s viability given the circumstances. Similar applications in research highlight the method’s utility in addressing logistical and ethical challenges in data collection [66, 67].

Due to the limitations of the convenience sampling method, data were collected over an extended period to mitigate common method bias (CMB). This approach aims to reduce the impact of systematic errors stemming from reliance on a single method by gathering data across different time periods [68]. The scales used in the study were presented to participants in a simple, clear, and specific manner, serving as one of the strategies to prevent responses from being influenced by systematic tendencies. These procedures enhance the validity of survey results and mitigate the effects of common method bias [66, 68]. Additionally, assuring participants of the confidentiality and anonymity of their responses is a widely accepted approach for mitigating social desirability bias in surveys [69]. When respondents are confident that their answers will not be linked to their identities, they are more likely to provide honest and accurate responses, free from concerns about social judgment. To the end, participants were assured that their responses would remain anonymous and confidential to mitigate the effects of social desirability bias.

An online questionnaire was used in this study. A web-based survey was employed for two primary purposes. First, it enables us to reach a wide audience of potential participants. Second, it provides a cost-effective and dependable means of collecting data [70]. The survey was disseminated to potential participants via the research team’s social media platforms. The online survey was created using Google Forms. The survey link was shared with participants via email, Facebook, Instagram, X (formerly Twitter), WhatsApp, and other social media platforms. Participants were first asked whether they had private health insurance. Those who answered "yes" were directed to proceed with the survey, while individuals without private health insurance were excluded from the study. The participants were informed that the research aimed to investigate the factors affecting the repurchase intention of private health insurance policy. The researchers shared questionnaire form on their social media profiles, and groups seeking assistance in recruiting volunteers for the study and encouraging insurance policyholders to participate. Throughout the data collection process, considerable emphasis was placed on ensuring the voluntary participation of respondents. The study was conducted in accordance with the 1964 Helsinki Declaration. The data were collected between 15.12.2023 and 15.03.2024. Informed consent was acquired from the participants, who were guaranteed the confidentiality and anonymity of their responses.

### Data analysis

The dataset contained no missing values, as participants were required to answer all questions. Descriptive statistics, including measures such as the mean, standard deviation (SD), skewness, and kurtosis, are presented. Construct validity, convergent validity, and discriminant validity were assured. Moreover, internal consistency reliability was tested via Cronbach's alpha coefficient. To investigate the relationships between the variables, the Pearson correlation coefficient was computed. Exploratory and confirmatory factor analyses were carried out to test the measurement model, and the hypothesized causal model between the variables was tested through structural equation modeling (SEM) via maximum likelihood estimation. The significance of the indirect effects was assessed using the bootstrapping method, which included 5,000 resamples to estimate the 95% confidence intervals (CIs). In the model, trust, perceived quality, customer satisfaction and repurchase intention were treated as latent variables. Data analysis involved testing several mediation models to examine the relationships between perceived quality, customer satisfaction, and other key variables. Different models were developed, including serial, parallel, and single mediation models, using structural equation modeling (SEM). Model fit was assessed using standard indices such as CFI, TLI, and RMSEA, while the statistical significance of the indirect effects was evaluated to determine the best-fitting model. The models were compared to identify the one that most accurately represented the data and provided the most meaningful insights into the mediating effects.

All the data were analyzed via SPSS version 25 and SPSS AMOS 24 for Windows. AMOS was preferred for its user-friendly interface and ability to perform complex Structural Equation Modeling (SEM) without requiring extensive programming knowledge. Its integration with SPSS, and widespread use in social sciences make it an ideal choice for analyzing the study’s data [71]. Despite the availability of other SEM software, such as LISREL or SmartPLS, AMOS was selected due to the research team’s familiarity and efficiency in using it, ensuring accurate and effective model testing and data analysis.

## Results

### Psychometric analysis

In this study, since the scales used had their validity and reliability evaluated for the first time by the authors in the Turkish and private health insurance sector literature, language validity was initially established. Following standard guidelines for cross-cultural adaptation, the original Quiet Quitting Scale (QQS) was translated into Turkish using the forward–backward translation method [72, 73]. This method ensures that the translated version retains the conceptual equivalence of the original scale and is culturally appropriate for the target population. After ensuring the language validity of the scales, exploratory factor analysis (EFA) was conducted separately for each scale. The trust scale is unidimensional, with factor loadings ranging from 0.77 to 0.89. The customer satisfaction scale has factor loadings between 0.88 and 0.92, while the perceived quality scale shows factor loadings ranging from 0.80 to 0.88. Finally, the repurchase intention scale has factor loadings between 0.91 and 0.94. These values indicate strong internal consistency for all scales, with each item’s factor loading reflecting a meaningful contribution to the construct being measured [74].

As Lt Hu and PM Bentler [75] suggested, construct validity is assured on the basis of CR and MaxR(H) > 0.7, convergent validity ensures AVE > 0.5, and discriminant validity is assured on the basis of HTMT < 0.9 thresholds. With maximum likelihood estimation, the confirmatory factor analysis (CFA) results indicated that the suggested one-factor solution for trust, perceived quality, customer satisfaction and repurchase intention was successfully replicated within this sample. On the basis of the CFA results, the model fit to the data was good (see the measurement model). Each item on the scales made a significant contribution to the corresponding factor, with standardized item factor loadings ranging from 0.69 to 0.94.

### Preliminary analysis

Preliminary analysis was conducted before the measurement and structural model were tested. For this purpose, we employed mean, standard deviation, correlation, skewness, and kurtosis values for each of the study variables. The potential issue of multicollinearity among the research variables was evaluated through correlation coefficients and Variance Inflation Factors (VIF) [76]. Correlation coefficients below 0.80, along with VIF values (from 2.86 to 3.28) less than 5, confirmed that multicollinearity was not a concern. The skewness and kurtosis values were evaluated via the guidelines proposed by George and Mallery [77] on the basis of the rule of thumb of ± 2. The results revealed that all the variables exhibited a relatively normal distribution (Table 2). Correlation analysis revealed that trust was positively correlated with perceived quality, customer satisfaction and repurchase intention. Perceived quality was positively correlated with customer satisfaction and repurchase intention. Furthermore, customer satisfaction was positively correlated with repurchase intention. On the other hand, the alpha coefficients and correlations between the study variables express the validity of the scales in terms of reliability and convergent and divergent validity (see Table 2).

**Table 2:** Mean score, standard deviation, Cronbach's alpha, and correlation coefficient for the study variables

	Mean	SD	α	1	2	3	4	Skewness	Kurtosis
1.Trust	3.49	0.83	0.90	1				-0.90	1.02
2.Perceived quality	3.59	0.91	0.97	.70**	1			-0.53	0.07
3.Customer satisfaction	3.85	0.92	0.93	.78**	.74**	1		-1.01	1.03
4.Repurchase Intention	3.58	0.87	0.94	.72**	.61**	.72**	1	-0.73	0.94

^**^p < 0.01; All correlations coefficients are Pearson's r

### Measurement model

Since no single statistic alone is adequate for determining the model’s goodness of fit, we utilized a variety of statistics, as suggested by Lt Hu and PM Bentler [75] and RB Kline [78], when evaluating the goodness of fit for both the measurement and structural models. Therefore, we employed a range of statistical measures, including the CMIN/DF (the chi-square to degrees of freedom ratio), root mean square error of approximation (RMSEA), standardized root mean square residual (SRMR), goodness-of-fit index (GFI), comparative fit index (CFI), and Tucker‒Lewis index (TLI), for assessing goodness of fit. In the measurement model, four interrelated latent variables, namely, trust, perceived quality, customer satisfaction and repurchase intention, are used. Trust was represented by a set of five items, perceived quality was represented by its five items, customer satisfaction was represented by a set of four items, and repurchase intention was represented by three items. A CMIN/DF value of less than 3 indicates a strong fit, and a value less than or equal to 5 indicates an acceptable fit. A model is deemed to exhibit a strong fit when the RMSEA and SRMR are both equal to or less than 0.06, whereas an acceptable fit is achieved if the RMSEA and SRMR are both equal to or less than 0.08. GFI, CFI, and TLI values of 0.95 or higher indicate a strong fit, and values of 0.90 or higher suggest an acceptable fit [79]. The results from the confirmatory factor analysis (CFA) indicated that the measurement model displayed a good fit with the data: χ2/df = 1.65, *p* < 0.001, TLI = 0.97, CFI = 0.97, RMSEA = 0.06, and SRMR = 0.03.

### Structural model

The study utilized structural equation modeling (SEM) with maximum likelihood estimation to assess the hypothesized connections among trust, perceived quality, customer satisfaction, and repurchase intention. The SEM findings indicated that the structural model was a good fit for the data, supported by various fit indices: χ2/df = 1.65, TLI = 0.97, CFI = 0.97, RMSEA = 0.06, and SRMR = 0.03. The suggested relationships between the variables are visually represented in Fig. 1. The Serial Mediation Model demonstrated the best fit among the models tested, with a CFI of 0.97, TLI of 0.97, and an RMSEA of 0.06, indicating a good model fit. The indirect effects in this model were statistically significant (*p* < 0.001), suggesting that both perceived quality and customer satisfaction serve as significant mediators in the relationship under investigation. The Parallel Mediation Model (Alternative Model 1) showed slightly weaker fit indices (CFI = 0.95, TLI = 0.94, RMSEA = 0.08), but still produced significant indirect effects (a*b* = *0.85, c*d = 0.01*). However, the Single Mediation Model (Perceived Quality), despite having excellent fit indices (CFI = 0.98, RMSEA = 0.04), did not yield statistically significant indirect effects (*p* > 0.05). Lastly, the Single Mediation Model (Customer Satisfaction) showed statistically significant indirect effects (*p* < 0.001), but its fit indices (CFI = 0.96, RMSEA = 0.09) were weaker than the other models (Table 3).

**Table 3:** Model fit indices for the tested mediation models

Model	CMIN	df	CFI	TLI	RMSEA	Indirect effect *p* value
Serial Mediation Model	185.420	112	0.97	0.97	0.06	0.00*
Parallel Mediation Model (Alternative model1)	251.336	114	0.95	0.94	0.08	a*b = 0.85 c*d = 0.01*
Single Mediation Model (Perceived Quality) (Alternative model2)	87.646	62	0.98	0.98	0.04	0.24
Single Mediation Model (Customer satisfaction) (Alternative model 3)	134.712	51	0.96	0.96	0.09	0.00*

On the basis of serial mediation model results, trust in the insurance company positively predicted perceived quality (β = 0.77, *p* < 0.001; supporting Hypothesis 1), customer satisfaction (β = 0.60, *p* < 0.001; supporting *Hypothesis 2*), and repurchase intention (β = 0.40, *p* < 0.005; supporting *Hypothesis 3*). Additionally, perceived quality positively predicted customer satisfaction quality (β = 0.34, *p* < 0.001; supporting *Hypothesis 4*), and customer satisfaction positively predicted repurchase intention quality (β = 0.44, *p* < 0.001; supporting *Hypothesis 5*). However, the direct effect of perceived quality on repurchase intention is not statistically significant (β = -0.02, *p* > 0.01; not supporting *Hypothesis 6*) (see Table 4). Furthermore, trust explained 59% of the variance in perceived quality. Together, trust and perceived quality explained 80% of the variance in customer satisfaction, and trust and customer satisfaction explained 68% of the variance in repurchase intention.

**Table 4:** Standardized and unstandardized direct estimates

Predictor	Outcome	B	β	S.E	*p*
Trust →	Perceived quality	0.77	0.77	0.07	< 0.001
Perceived quality →	Customer satisfaction	0.37	0.34	0.08	< 0.001
Customer satisfaction →	Repurchase Intention	0.43	0.44	0.11	< 0.001
Trust →	Customer satisfaction	0.66	0.60	0.08	< 0.001
Trust →	Repurchase Intention	0.43	0.40	0.13	< 0.001
Perceived quality →	Repurchase Intention	-0.03	-0.02	0.10	> 0.01

The mediating effect of perceived quality and customer satisfaction significance was examined through the bootstrap estimation method in AMOS 24. To this end, on the basis of previous suggestions KJ Preacher and AF Hayes [80], a total of 5000 bootstrap samples were generated through random sampling with replacement from the original dataset to estimate the 95% confidence interval (CI). As hypothesized, the indirect effect of trust on customer satisfaction via perceived quality was significant and positive (β = 0.29, SE = 0.07, 95% CI [0.14, 0.46], supporting *Hypothesis 7*. The indirect effect of trust on repurchase intention via customer satisfaction was significant and positive (β = 0.29, SE = 0.10, 95% CI [0.10, 0.51], supporting *Hypothesis 8*. Additionally, the present study assessed the serial mediating role of perceived quality and customer satisfaction in the relationship between trust and repurchase intention. The findings indicate that the indirect effect of trust on repurchase intention through perceived quality and customer satisfaction is significant (β = 0.12, SE = 0.05, 95% CI 0.03, 0.24), supporting *Hypothesis 9* (see Table 5).

**Table 5:** Standardized indirect effects

					95% CI indirect effect	
Predictor	Mediator	Outcome	β	S.E	Lower Bound	Upper Bound
Trust →	Perceived quality →	Customer satisfaction	0.29	0.07	0.14	0.46
Trust →	Customer satisfaction →	Repurchase Intention	0.29	0.10	0.10	0.51
Trust →	Perceived quality → Customer satisfaction →	Repurchase Intention	0.12	0.05	0.03	0.24

## Discussion

The aim of the present study is to investigate the mediating role of perceived quality and customer satisfaction in the relationship between trust and repurchase intentions in the private health insurance sector. There are studies in the literature indicating the impact of trust, perceived quality, and customer satisfaction on repurchase intention. As trust, satisfaction, and perceived quality increase, the intention to repurchase also increases [22, 33, 35, 37, 42].

The first three hypotheses assert that trust is significantly related to perceived quality, customer satisfaction and repurchase intention. The findings obtained from the present study have shown that trust has a direct effect on perceived quality. Moreover, trust has a direct effect on customer satisfaction. Furthermore, trust had a significant effect on repurchase intentions.

In parallel with our first hypothesis, a positive relationship between trust and perceived quality was found [81]. When trust in the insurance company is greater, perceived quality is expected to be greater. Additionally, perceived service quality significantly impacts customer satisfaction, which in turn influences trust and loyalty in e-commerce settings [37]. In another study, perceived quality had a positive direct effect on foreign brand trust [82]. In parallel with our findings, trust in an insurance company plays a crucial role in influencing how customers perceive the quality of the services offered [37]. When policyholders trust their insurance provider, they are more likely to view the quality of the services as superior, which, in turn, enhances their overall satisfaction. For the second hypothesis, we investigated whether trust is significantly related to customer satisfaction. The results revealed a significant and direct effect of trust on customer satisfaction. An empirical investigation suggested that trust is the most important factor influencing customer satisfaction in insurance firms, followed by value, empathy, and resistance to change [83]. Jang [84] and Miao et al. [85] found that the feeling of trust plays a significant role in repurchase intention and customer loyalty. Similar with our study suggested, the findings from other studies on the second hypothesis have yielded similar results [86, 87]. The third hypothesis investigated the impact of trust on repurchase intention. We found a direct and significant effect of trust on repurchase intentions. The literature suggests that trust and satisfaction are determinants of repurchase intention in parallel with our findings [88]. Trust is a significant determinant of repurchase intention, and studies in the literature support this finding [89–93]. Moreover, increasing satisfaction, perceived quality, and trust positively influence repurchase intentions in online group buyers, as in health insurance owners [90]. For the fourth hypothesis, we investigated whether perceived quality was significantly related to customer satisfaction. The findings suggested that perceived quality had a direct and significant effect on customer satisfaction. U Šebjan and P Tominc [94] reported that perceived fair insurance services had a significant positive effect on customer satisfaction. Additionally, a study conducted in the insurance sector reported that perceived service quality affects the satisfaction and loyalty of bank account managers with insurance companies [95]. This result concurs with the research suggesting that perceived quality is significantly related to customer satisfaction [96]. The fifth hypothesis examined whether customer satisfaction was significantly related to repurchase intention. The findings indicate that customer satisfaction significantly and positively affects repurchase intention. The effect of customer satisfaction on repurchase intentions has been examined in many studies, and the results obtained from these studies are similar to our findings [50–53]. When satisfied with private health insurance policy, customers tend to repurchase more [52]. Hypothesis 6 examined whether perceived quality is significantly related to repurchase intentions. On the basis of analysis results there was no significant relationship. Studies in the literature suggested that perceived quality significantly impact repurchase intention. This divergence can be attributed to the unique dynamics of the private health insurance sector, which may influence customers’ perceptions. Factors such as intense competition, standardized services, or limited awareness could diminish the direct impact of perceived quality on repurchase intention. Additionally, contextual factors such as the timing of the study, prevailing economic conditions, or specific policies within the insurance industry might have weakened the relationship between perceived quality and repurchase intention. Furthermore, in the context of the private health insurance sector, the non-significant direct effect of perceived quality on repurchase intention could be attributed to several industry-specific dynamics. One possible explanation is that customers' decision-making processes in health insurance are influenced more by factors such as trust, service reliability, and customer support rather than solely by perceived quality. Additionally, other mediating variables, such as customer experience with claims processing or the overall perceived value of the insurance plan, may play a more prominent role in shaping repurchase intentions. Sector-specific factors, such as regulatory influences, competition, and the long-term nature of insurance contracts, might also mitigate the direct impact of perceived quality, making trust and satisfaction more significant predictors of repurchase intention in this particular context. However, rather than a direct relationship, the link between perceived quality and repurchase intention may be better explained through another variable, such as customer satisfaction, acting as a mediator.

In the last 3 hypotheses, we investigated the mediating role of perceived quality in the relationship between trust and satisfaction (*Hypothesis 7)* and the mediating role of customer satisfaction in the relationship between perceived quality and repurchase intention (*Hypothesis 8*) and the serial mediating (*Hypothesis 9*) roles of perceived quality and customer satisfaction in the relationship between trust and repurchase intention. The indirect effect of trust on customer satisfaction via perceived quality was significant and positive. N Kassim and N Asiah Abdullah [37] stated that perceived service quality positively impacts customer satisfaction and trust. In the present study, we concluded that there is a significant effect of trust on customer satisfaction and that perceived quality acts as a mediator. The indirect effect of trust on repurchase intention via customer satisfaction was significant and positive. Consistent with our findings, MA Saleem et al. [34] reported that trust directly impacts repurchase intentions in the Pakistani airline industry, with customer satisfaction acting as a mediator. In other studies, customer trust and satisfaction positively influence repurchase intentions for e-retailers, with the price level mediating this relationship [97]. A Akhondi and A Kafashpor [98] and Ginting et al. [99] reported that trust positively influences repurchasing, with customer satisfaction acting as a mediator, in parallel with our findings. Kusumo et al. [96] indicated that customer satisfaction acts as mediator variable in the relationship between service quality with repurchase intention in parallel with our findings. In the last hypothesis, we examined the indirect effect of trust on repurchase intention through perceived quality, and customer satisfaction was significant. According to the findings, perceived quality and customer satisfaction play a serial mediating role in the relationship between trust and repurchase intention. L Su et al. [60] reported that perceived service quality positively impacts repurchase intentions and that customer satisfaction acts as a mediator. Additionally, A Agyapong et al. [100] reported that perceived service quality positively impacts patients’ satisfaction and behavioral intentions in healthcare, with satisfaction acting as a mediator between service quality and patient behavior. Studies in the literature have revealed significant results for perceived quality and customer satisfaction as mediating variables, but to the best of our knowledge, the serial mediating role of these two variables in the insurance sector is examined for the first time in this study. Therefore, this study is expected to make important contributions to the relevant literature.

### Contribution and implications of the findings

This research examines the impact of trust, perceived service quality, and customer satisfaction on the intention to repurchase a private health insurance policy. Additionally, the study investigates the mediating effect of perceived quality and customer satisfaction on the relationship between trust and the intention to repurchase. The current study contributes to the existing health insurance literature by examining the influence of trust in insurance companies, the perceived quality of services, and customer satisfaction on the repurchase intentions of policies in the context of insurance services. Additionally, this study makes significant contributions to the literature on theories related to repurchase intention. According to the theory of status quo bias, consumers tend to avoid the risks associated with changing or switching service providers. When insurance policyholders trust their insurance company, appreciate the quality of services, and feel satisfied, they may be more inclined to renew their policies, as posited by the theory. Regarding the status quo bias, the results support the theory by demonstrating that trust, satisfaction, and perceived quality contribute to a consumer’s intention to repurchase their health insurance policy, reflecting the tendency to stick with the current provider due to a preference for avoiding the risks associated with change. The finding that repurchase intention is strongly influenced by trust and satisfaction reinforces the idea that consumers are more likely to renew policies when they feel secure and satisfied with their current provider, consistent with the status quo bias theory.

On the other hand, the disconfirmation theory indicates that consumers' expectations and experiences influence their satisfaction and perceptions of a product or service. The research findings demonstrated that satisfied insurance policyholders are more willing to repurchase their insurance policies, thereby supporting the theory. For disconfirmation theory, the study highlights that satisfaction and repurchase intention are shaped by the alignment (or misalignment) between customer expectations and their experiences. Although perceived quality did not have a direct effect on repurchase intention, the satisfaction with the insurance services reflects how well expectations are met, supporting the disconfirmation theory. This indicates that customers are likely to renew their policies if their experiences align with their expectations, which directly affects their satisfaction levels and ultimately influences repurchase decisions. Additionally, our study makes a significant contribution to the existing research on consumer behavior in the health insurance sector. While the current literature generally focuses on key factors such as trust, satisfaction, and perceived quality, this study provides a deeper perspective by examining the relationships between trust, perceived quality, customer satisfaction, and repurchase intention among private health insurance owners. Previous studies have mainly focused on the direct relationship between trust and customer satisfaction. In contrast, this study employs perceived quality and customer satisfaction as serial mediating variables, highlighting the role of these factors in the relationship between trust and repurchase intention. Furthermore, SEM used in our study allows for a more comprehensive examination of this relationship, showing how consumer behavior develops as a multi-stage and dynamic process, which differentiates this study from existing research. By emphasizing the serial mediating roles of trust, perceived quality, and satisfaction in the health insurance sector, this study offers a novel perspective compared to current literature. In this context, our findings help address gaps in understanding consumer behavior in the health insurance sector and provide a deeper explanation of the various dynamics within the industry. The results of this study provide significant contributions by evaluating and comparing various models to better understand the mediating mechanisms between key variables. The Serial Mediation Model was found to be the most robust, offering a comprehensive framework that simultaneously considers both perceived quality and customer satisfaction as significant mediators. With strong fit indices and statistically significant indirect effects, this model appears to be the most reliable for capturing the complexities of these relationships. The Parallel Mediation Model, though providing meaningful indirect effects, showed weaker fit indices, suggesting that it is less robust than the serial model. Additionally, the Single Mediation Models, while demonstrating excellent fit in the case of Perceived Quality, did not produce statistically significant indirect effects. These findings emphasize the importance of comparing alternative and nested models to ensure that the selected model provides the best representation of the data. The Serial Mediation Model is not only the best fitting but also offers more insights into how perceived quality and customer satisfaction interact, offering practical implications for insurance companies aiming to improve repurchase intention by considering these factors together.

Private health insurance in Türkiye is developing rapidly, and new insurance companies are emerging. For this reason, it is not easy to retain the existing customer portfolio. To the best of our knowledge, this study on the factors affecting repurchase intentions in the health insurance literature is the first study conducted in Türkiye, and no similar study has been reported in the international literature. It is thought that this research will make significant contributions to the literature. When the findings are evaluated, insurance companies can have an idea about whether the offered policy will be purchased again by customers.

While this study may provide direct guidance to insurance companies in practice, it also indirectly contributes significantly to the improvement of individuals’ health in society. Healthcare services are sustained not only in Türkiye but also in many countries worldwide through the presence of the private sector alongside the public sector. The role of the private healthcare sector is crucial in reducing the burden on public healthcare services. The easiest way to receive private healthcare services without facing catastrophic healthcare expenditures is through private health insurance. Therefore, increasing the number of private health insurance policies holds a significant place, contributing to both the country’s economic development and the enhancement of the public’s health status.

To enhance practical implications in the context of private health insurance, addressing industry-specific challenges is crucial. Insurers should focus on alleviating customer pain points, such as improving access to healthcare services and streamlining claim processes. Leveraging digital tools, such as telemedicine platforms and online policy management systems, can significantly improve perceived quality and customer satisfaction. Additionally, offering personalized insurance plans tailored to individual health needs and providing proactive communication about policy coverage can foster trust and enhance customer loyalty. Expanding the network of healthcare providers and ensuring transparency in policy terms further contribute to a higher perceived value and improved customer retention. Moreover, the use of well-known and popular public figures as brand ambassadors in advertisements by insurance companies can also enhance trust in these companies and positively contribute to customers’ repurchase intentions. These industry-specific recommendations can help insurers better meet customer expectations and improve their competitive positioning.

## Limitations

Similar to other studies, the present research has specific limitations. First, a convenience sampling method was utilized, which may restrict the generalizability of the findings. On the other hand, the study was conducted in a specific city in Türkiye, and thus generalizing the results to the entire population of Türkiye may not be warranted. It would be advantageous to replicate and expand upon this study using diverse samples to enhance the applicability of the findings. Second, the research was designed as a cross-sectional study, which limits its ability to establish causal relationships between the variables. The use of convenience sampling method and cross-sectional design present significant threats to validity and generalizability in research. Particularly, CMB emerges as a major limitation in such studies. Despite efforts by researchers to control for CMB, it cannot be entirely eliminated due to the inherent constraints of self-report surveys, the use of convenience sampling, and the application of cross-sectional designs. These factors contribute to systematic errors, as they often involve a single method or time point for both the predictor and outcome measures, leading to inflated or distorted relationships between variables. While, participants were assured that their responses would remain anonymous and confidential to mitigate the effects of social desirability bias, the risk associated with CMB cannot be completely eliminated due to the use of self-report questionnaires. Even though efforts are made to control for this bias, its complete removal remains challenging, especially when self-assessment tools, convenience sampling, and cross-sectional designs are utilized. Third, the cross-sectional design of the study limits the ability to draw causal conclusions between variables. This approach only provides a snapshot of the relationship between variables, making it challenging to assess the direction of influence or any potential cause-and-effect links over time. However, İstanbul is the city with the highest internal migration in Türkiye, and its sample can provide valuable insights into the general characteristics and attitudes of the Turkish population. This can be considered a potential solution to the issue of data generalizability. Nevertheless, to support the generalizability of the findings, longitudinal studies are needed, as they allow for the observation of changes and the assessment of causal relationships over an extended period. Lastly, the study focused solely on the indirect effects of trust on repurchase intentions through only two mediating variables. The literature suggests that other factors may also influence repurchase intentions. Future research should consider the impacts of customer loyalty, switching costs associated with insurance, preferences for specific companies, and the financial status of policyholders.

Although the limitations section acknowledges the use of a convenience sampling method and cross-sectional design, a more thorough examination of the implications of these methodological limitations is warranted. The use of a convenience sample restricts the generalizability of the findings, particularly in terms of extending the results to broader populations beyond the specific region of İstanbul. To address this limitation, future research could expand the sample to include more diverse regions across Türkiye, or even internationally, to increase the external validity of the results. Additionally, the cross-sectional design limits the ability to draw causal inferences. Longitudinal studies would be beneficial to examine the direction of causality and the long-term effects of trust, satisfaction, and perceived quality on repurchase intentions. Future research could also explore the effects of other potential mediators, such as customer loyalty, switching costs, or financial factors, which were not included in the current study but may significantly impact repurchase intentions.

## Conclusion

Considering our comprehensive findings, we can infer that our study model effectively elucidated the hypothesized causal relationships among all the independent and dependent variables. All hypotheses in the present study have been confirmed, as mentioned in the results section. In the first three hypotheses, we examined whether trust is significantly related to perceived quality, customer satisfaction and repurchase intention. All the variables had a significant and positive effect on repurchase intentions. For the fourth hypothesis, we investigated whether perceived quality was significantly related to customer satisfaction and found that perceived quality had a significant and positive effect on customer satisfaction. Additionally, we also found that customer satisfaction was significantly related to repurchase intention. In the last three hypotheses, we investigated the mediating role of perceived quality in the relationship between trust and satisfaction, and we found a significant and positive effect. Moreover, the mediating role of customer satisfaction in the relationship between perceived quality and repurchase intention was positive and significant. The serial mediating roles of perceived quality and customer satisfaction in the relationship between trust and repurchase intentions were statistically significant.

Private health insurance providers should prioritize fostering and maintaining customer trust, as it plays a critical role in shaping perceived quality and customer satisfaction, which ultimately influence repurchase intentions. To achieve this, insurers should emphasize clear and open communication, showcasing the value and benefits of their offerings to strengthen consumer trust. In addition, enhancing the perceived quality of their services—such as by providing comprehensive coverage options and delivering exceptional customer support will be key to boosting customer satisfaction and cultivating lasting relationships. Furthermore, insurance companies should continuously assess customer satisfaction through regular surveys and feedback mechanisms, ensuring any issues or concerns are addressed swiftly. Given that customer satisfaction acts as a mediator between trust and repurchase intention, focusing on enhancing satisfaction levels can have a direct impact on customer retention and increase the likelihood of policy renewals. By adopting these strategies, private health insurance firms can improve customer loyalty while also ensuring their continued profitability in a competitive market landscape.

İstanbul is Türkiye’s most cosmopolitan city. It receives internal migration from all 80 provinces of Türkiye and is also the country’s most populous city. According to the Internal Migration Statistics Report by the İstanbul Planning Agency (IPA), İstanbul remained the leading province in internal migration in 2023, with 412,707 people moving to the city [101]. Therefore, this study conducted in İstanbul sample. The İstanbul sample can provide insights into the general characteristics and attitudes of the Turkish population. To assess regional differences, studies could be conducted in the provinces with the highest number of private health insurance holders in each region of Türkiye, enabling a nationwide generalization.

## Supplementary Information

Supplementary Material 1.

## Data Availability

The data supporting this study’s findings are available from the corresponding author, [İG], upon reasonable request.
