# Supplementary material for: The serial mediation effect of perceived quality and customer satisfaction on the relationship between trust and repurchase intention: a research on private health insurance owners
Source: BMC Health Serv Res. 2025 Feb 15;25:257. doi: 10.1186/s12913-025-12269-9 (PMC11830212; doi:10.1186/s12913-025-12269-9)
Supplement: Supplementary file 1 — Supplementary Material 1. [file 12913_2025_12269_MOESM1_ESM.docx]

**Appendix A**

**English Version of Scale Items**

|  | Perceived quality scale | 1. Strongly disagree | 2. Disagree | 3.Undecided | 4. Agree | 5. Strongly Agree |
| --- | --- | --- | --- | --- | --- | --- |
| 1 | The company employees inform me exactly when healthcare services will be provided. | 1 | 2 | 3 | 4 | 5 |
| 2 | The company employees deliver their services without any delays. | 1 | 2 | 3 | 4 | 5 |
| 3 | The company employees are always willing to assist me. | 1 | 2 | 3 | 4 | 5 |
| 4 | The behavior of the company employees instills trust in me. | 1 | 2 | 3 | 4 | 5 |
| 5 | The company employees understand my special requests. | 1 | 2 | 3 | 4 | 5 |
|  |  |  |  |  |  |  |
|  |  |  |  |  |  |  |
|  |  |  |  |  |  |  |
|  |  |  |  |  |  |  |
|  | Trust Scale | 1. Strongly disagree | 2. Disagree | 3.Undecided | 4. Agree | 5. Strongly Agree |
| 1 | Based on my past experiences, I know that the company from which I purchased my policy is honest. | 1 | 2 | 3 | 4 | 5 |
| 2 | Based on my past experiences, I know that my company cares about its policyholders. | 1 | 2 | 3 | 4 | 5 |
| 3 | Based on my past experiences, I know that the company does not engage in opportunistic behavior. | 1 | 2 | 3 | 4 | 5 |
| 4 | Based on my past experiences, I know that the company fulfills its commitments to policyholders. | 1 | 2 | 3 | 4 | 5 |
| 5 | Based on my past experiences, I know that the company is trustworthy. | 1 | 2 | 3 | 4 | 5 |

|  |  |  |  |  |  |  |
| --- | --- | --- | --- | --- | --- | --- |
|  | Customer Satisfaction Scale | 1. Strongly disagree | 2. Disagree | 3.Undecided | 4. Agree | 5. Strongly Agree |
| 1 | I believe that my decision to purchase the insurance policy was a wise one. | 1 | 2 | 3 | 4 | 5 |
| 2 | I feel good about my decision to obtain the insurance policy from the company. | 1 | 2 | 3 | 4 | 5 |
| 3 | I am satisfied with the insurance policy I purchased from this company. | 1 | 2 | 3 | 4 | 5 |
| 4 | I would recommend obtaining a health insurance policy from this company to others. | 1 | 2 | 3 | 4 | 5 |
